# Oral supplementation with areca-derived polyphenols attenuates food allergic responses in ovalbumin-sensitized mice

**DOI:** 10.1186/1472-6882-13-154

**Published:** 2013-07-02

**Authors:** Chia-Chi Wang, Yu-Ru Lin, Mei-Hsiu Liao, Tong-Rong Jan

**Affiliations:** 1 School of Pharmacy, Kaohsiung Medical University, No. 100, Shih-Chuan 1st Road, 807, Kaohsiung, Taiwan; 2 Department and Graduate Institute of Veterinary Medicine, School of Veterinary Medicine, National Taiwan University, No. 1, Section 4, Roosevelt Road, 106 Taipei, Taiwan; 3 Radiopharmaceuticals Production and Marketing Center, Institute of Nuclear Energy Research, No. 1000 Wenhua Road, Jiaan Village, 325, Longtan Township, Taoyuan, Taiwan

**Keywords:** Areca Nut, Food Allergy, Mast Cell, Myeloid-derived Suppressor Cells, Ovalbumin, Polyphenol

## Abstract

**Background:**

*Arecae semen*, the dried slice of areca nuts, is a traditional Chinese medicine used to treat intestinal parasitosis, rectal tenesmus and diarrhea. Areca nuts contain a rich amount of polyphenols that have been shown to modulate the functionality of mast cells and T cells. The objective of this study is to investigate the effect of polyphenol-enriched areca nut extracts (PANE) against food allergy, a T cell-mediated immune disorder.

**Methods:**

BALB/c mice were left untreated or administered with PANE (0.05% and 0.1%) via drinking water throughout the entire experiment. The mice were sensitized with ovalbumin (OVA) twice by intraperitoneal injection, and then repeatedly challenged with OVA by gavage to induce food allergic responses.

**Results:**

PANE administration attenuated OVA-induced allergic responses, including the occurrence of diarrhea and the infiltration and degranulation of mast cells in the duodenum. The serum level of OVA-specific IgE and the expression of interleukin-4 in the duodenum were suppressed by PANE treatment. In addition, PANE administration induced Gr-1^+^, IL-10^+^ and Gr-1^+^IL-10^+^ cells in the duodenum.

**Conclusion:**

These results demonstrate that oral intake of areca-derived polyphenols attenuates food allergic responses accompanied with a decreased Th2 immunity and an enhanced induction of functional myeloid-derived suppressor cells.

## Background

*Areca catechu* Linn., a member of the *Palmaceae* family widely cultivated in South-Asian countries, is a folk medicine employed to ameliorate symptoms of gastrointestinal inflammation, dysentery and diarrhea [1,2]. Dried areca nuts, including its slices (*Arecae semen*) and pericarp (areca peel; Da Fu Pi), are traditional Chinese medicines used to treat distended abdomen and intestinal parasitosis, tenesmus and diarrhea [1,3]. Although this herb has a long history of medicinal use, limited evidence is available to substantiate its claimed pharmacological effects.

Areca nuts contain alkaloids, carbohydrates, crude fiber, fats, proteins and polyphenols that are structurally similar to grape seed- and apple-derived procyanidins [4-6]. Polyphenols have been shown to exhibit a broad spectrum of biological activities, including antioxidant, anti-inflammatory, anti-allergic and immunomodulatory effects [7-9]. Accumulating evidence suggests that areca nuts possess a variety of potential pharmacological activities. Of relevant to this study, areca nut extracts inhibited antigen-induced degranulation in RBL-2H3 mast cells and the functionality of murine splenic lymphocytes and neutrophils [10-13]. Oral administration with a hydroalcoholic extract of areca nuts prevented nitroglycerine infusion-induced inflammatory responses and induced analgesic and anti-inflammatory effects in a murine hot plate model [14,15]. In addition, feeding rats with areca-derived polyphenols suppressed carrageenan-induced inflammatory responses and prostaglandin E_2_ formation [16]. These reports indicate the anti-inflammatory and immunomodulatory potential of areca nut constituents. However, it remains unclear if areca-derived polyphenols affect food allergic responses.

**Figure 1:**
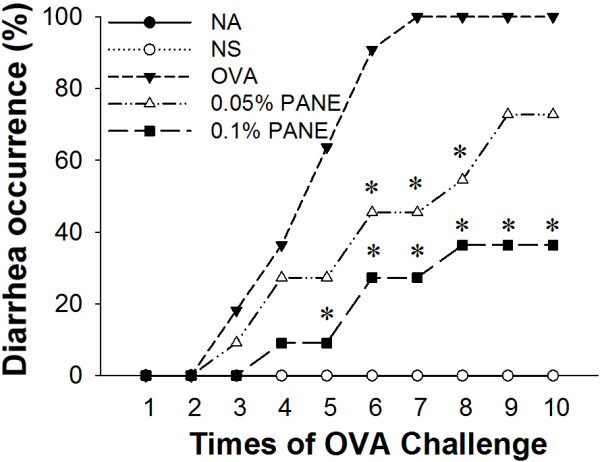
**PANE attenuated the occurrence of allergic diarrhea.** Mice were treated as the protocol described in the Methods section. Allergic diarrhea was monitored visually for 3 h after OVA challenge. Mice with profuse liquid stool were identified as diarrhea-positive animals. Data are pooled from 3 independent experiments (n = 11 per group). ^*^, *p* < 0.05 compared to the OVA group.

Food allergy is an immune disorder to dietary proteins affecting up to 6% of the population in developed countries [17]. Eggs, milk, peanuts, seafood and wheat are the most common allergens known to cause food hypersensitivity [18]. Symptoms associated with food allergy may range from mild gastrointestinal discomfort to severe and life-threatening anaphylactic shock. The immunological mechanisms of food allergy require the processing and presentation of allergens by antigen-presenting cells to T helper (Th) cells that subsequently differentiate to the Th2 phenotype. The Th2 signature cytokine IL-4 plays a pivotal role in the pathophysiology of allergy via the induction of IgE synthesis and mast cell proliferation [19-21]. To date, avoidance of allergens is the principal way to manage food allergy. Pharmacotherapy is employed solely for relieving hypersensitivity symptoms. Hence, the development of effective strategies to prevent or treat this disorder is of great importance.

As areca nut extracts have been shown to affect the functionality of mast cells and lymphocytes that are key players involved in the pathophysiology of type I hypersensitivity, we hypothesize that areca-derived polyphenols may be effective in modulating food allergic responses. We report here the anti-allergic effect of areca-derived polyphenols administered via drinking water in a murine model of food allergy.

## Methods

### Reagents and antibodies

All reagents were purchased from Sigma (St. Louis, MO) unless otherwise stated. Cell culture medium and fetal bovine serum (FBS) was from Hyclone (Logan, UT). Anti-mouse IL-4 rat IgG_1_, anti-mouse Gr-1 rat IgG_2b_ and anti-mouse IL-10 rat IgG_2b_ were from BioLegend (San Diego, CA). The anti-rat IgG secondary antibodies conjugated with alkaline phosphatase (AP) or horse radish peroxidase (HRP) were from AbCam, Inc. (Cambridge, UK).

### Plant material and extraction

Fresh tender nuts of *Areca catechu* Linn. were directly purchased from farmer, Chiayi County, Taiwan, in March, 2009. The nuts were identified by Dr. Ih-Sheng Chen (School of Pharmacy, Kaohsiung Medical University) by comparison with a voucher specimen deposited at the Herbarium of the Department of Botany of National Taiwan University (No: TAI 152786, collected and identified by Chien-Chang Hsu on Aug 26, 1969). Polyphenol-enriched areca nut extracts (PANE) were prepared as previously described [16]. Briefly, after removing the husks, the nuts were extracted three times with 80% acetone (1:10 w/v) and filtrated. The filtrate was evaporated to remove acetone, partitioned with n-hexane and ethyl-ether to remove lipids and then freeze-dried. The yield of the PANE extraction was 16%. The amount of condensed tannins in PANE was > 95% as measured by the acidified vanillin method previously described [22]. The level of endotoxin in the PANE was below the detection limit (0.05 endotoxin unit/mL) using an assay kit (Kinetic-QCL®; Lonza Walkersville Inc., Walkersville, MD).

### Protocol of animal experiments

Male BALB/c mice, 5–6 weeks of age, were purchased from the Animal Breeding Center of the National Taiwan University Hospital (Taipei, Taiwan). The mice were randomized, transferred to plastic cages containing a saw-dust bedding (4–5 mice per cage) and housed in a temperature (23 ± 2°C), humidity (60 ± 20%) and light (12-h light/dark cycle)-controlled environment. The present study employed a murine model of food allergy previously described [23]. In brief, mice were randomly divided into the following groups: naïve (NA), nonsensitized (NS), OVA-sensitized and challenged (OVA), and OVA-sensitized and challenged and PANE-treated (PANE). Mice received PANE via drinking water containing 0.05 and 0.1% (w/v) throughout the entire treatment period. The dosing regimen was chosen according to previous reports showing the immunomodulatory activity of apple polyphenols [9] and the anti-inflammatory, hepatoprotective and immunomodulatory activities of areca nut extracts [14-16,24]. Except for the NA and NS groups, mice were sensitized with OVA by intraperitoneal injection using 0.1 mL sensitization solution containing 50 μg OVA and 1 mg alum on day 3 and boosted with a double dose on day 17. Serum of mice was collected prior to OVA challenge on day 31. To induce allergic responses, mice were repeatedly challenged with OVA (50 mg/0.3 mL in saline/mouse) by gavage every other day from day 31 to day 49. Allergic diarrhea characterized as profuse liquid stool was monitored visually for 3 h after challenge. All mice were euthanized 3 h after the last OVA challenge and the spleen and duodenum were harvested for further experimentation. The animal experiments were approved by the Institutional Animal Care and Use Committee of the National Taiwan University.

### Spleen index

The spleen of each mouse was dissected out and weighed immediately after sacrifice. The spleen index was calculated as the spleen weight (mg) per body weight (g).

### Cellularity of splenocytes

Splenocytes were stained with rat anti-mouse CD4 and Gr-1 conjugated with FITC, and rat anti-mouse CD8, CD11b, B220 conjugated with PE-Cy5 antibodies (BioLegend, San Diego, CA) in PBS containing 2% FBS. After washing, the single cell fluorescence of 10,000 cells for each sample was measured by a flow cytometer (BD FACSCalibur, San Jose, CA). Data were analyzed using the software Flowjo 5.7.

### Histological examination of duodenum

The duodenum was excised and fixed in 10% neutral buffered formalin for 2 days. Tissue were embedded in paraffin, sectioned at a thickness of 4–5 μm and stained with hematoxylin and eosin (H&E) for routine histopathology. The ratio of villi length over crypt depth was measured by Image-Pro Plus 5.1 morphometric analysis software (Media Cybernetics, Inc. Rockville, MD). Tissue sections were also stained with toluidine blue for identification of mast cells. The number of total and degranulated mast cells was counted manually.

### Enzyme-linked immunosorbent assay (ELISA) for antibody measurement

The levels of total and OVA-specific IgE in serum samples were measured by ELISA [25].

### Immunohistochemical staining

Tissue sections were deparaffinized and then rehydrated following a standard procedure. The rehydrated slides were immersed in Trilogy™ (Cell Marque, Hot Springs, AR) at 121°C for 15 min for antigen retrieval. The endogenous peroxidase activity was then quenched with 3% H_2_O_2_ in methanol and blocked with normal goat serum. Primary antibodies were applied onto each section overnight. The slides were treated with super enhancer, and then incubated with poly-HRP reagent. For visualization, the slides were treated with the peroxidase substrate 3-amino-9-ethylcarbazole (AEC) for 2 min followed by hematoxylin counter staining (blue color). For IHC double staining, AEC-treated slides were incubated with another primary antibody at 4°C in the dark overnight followed by incubation with AP-conjugated secondary antibody for 1 h. The slides were then treated with AP substrate, 5-bromo-4-chloro-3-indolyl phosphate/nitroblue tetrazolium (BCIP/NBT), for 30 min for observation of a second staining without counter-staining. The number of IHC-positive signals was quantified using the Image Pro Plus 5.1 program. The number of double positive cells showing dark blue surrounded with red color was counted manually. Six duodenums per group were analyzed at 200-fold magnification.

### Statistical analysis

Data of diarrhea occurrence were expressed as percentage and analyzed by the Chi-square test to compare the difference between PANE-treated groups and the OVA control. All other data are expressed as the mean ± standard error (SE) for each treatment group in the individual experiments. Normality and homoscedasticity of data were tested by the Shapiro-Wilk test and the Bartlett’s test, respectively. Analysis of variance was performed by one-way ANOVA. Dunnett’s two-tailed *t-*test was used to compare treatment groups to the control group. *P* value < 0.05 was defined as statistical significance.

## Results

### PANE did not affect body weight, and the spleen index and cellularity

We firstly investigated whether administration of PANE via drinking water influenced the spleen index and cellularity in mice with food allergy. As shown in Table 1, oral intake of PANE (0.05% and 0.1%) did not affect the body weight, the spleen index and the population of splenic CD4^+^, CD8^+^, B220^+^ and CD11b^+^ cells.

**Table 1:** No effect of PANE on the body weight, and the spleen index and cellularity in mice with food allergy

	**NA**	**NS**	**OVA**	**PANE**	
				**0.05%**	**0.1%**
**Body weight (g)**					
Day 1	24.5 ± 0.8	24.1 ± 0.7	23.9 ± 0.5	25.0 ± 0.7	24.4 ± 0.6
Day 49	28.6 ± 0.5	27.5 ± 0.5	27.4 ± 0.4	27.4 ± 0.7	27.1 ± 0.5
**Spleen weight (mg)**	88.4 ± 3.5	84.9 ± 4.5	91.0 ± 2.8	87.6 ± 3.6	93.0 ± 2.7
**Spleen index**	3.0 ± 0.1	3.0 ± 0.1	3.2 ± 0.1	3.1 ± 0.1	3.3 ± 0.1
**Spleen cellularity (%)**					
CD4^+^	26.4 ± 0.2	26.7 ± 0.8	25.6 ± 1.2	25.7 ± 1.3	26.0 ± 1.4
CD8^+^	10.6 ± 0.4	11.0 ± 0.6	11.0 ± 0.3	11.5 ± 0.6	10.6 ± 0.5
B220^+^	55.1 ± 1.5	55.2 ± 1.9	55.8 ± 3.5	55.5 ± 2.6	56.6 ± 3.3
CD11b^+^	4.2 ± 0.4	4.9 ± 0.2	4.3 ± 0.1	4.3 ± 0.1	4.4 ± 0.3

**Figure 2:**
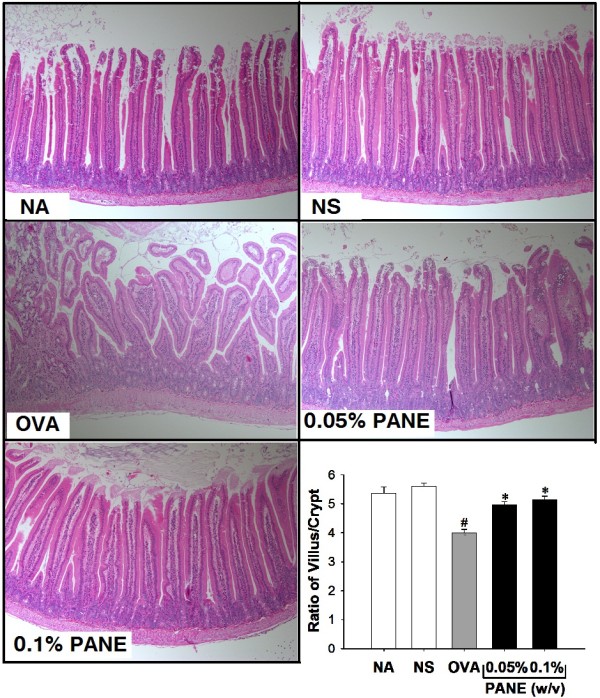
**PANE attenuated the infiltration of inflammatory cells and crypt hyperplasia in the duodenum of OVA-sensitized and challenged BALB/c mice.** Mice were treated as the protocol described in the Methods section. Representative sections of the duodenum stained with H&E were shown (original magnification, ×100). Six measurements per section were counted and the villus/crypt ratio was measured. Data are expressed as mean ± SE of 11 mice per group pooled from 3 independent experiments. ^#^, *p* < 0.05 compared to the NS group. ^*^, *p* < 0.05 compared to the OVA group.

Mice were treated with PANE as described in Figure 2. The spleen index was calculated as the spleen weight (mg) per body weight (g). Splenocytes were stained for CD4, CD8, B220 and CD11b and analyzed by flow cytometry. Data are expressed as mean ± SE of 11 samples per group pooled from 3 independent experiments.

### PANE attenuated the occurrence of allergic diarrhea

We next investigated the effect of PANE on OVA challenge-induced diarrhea that is a hallmark of food allergic reactions. Repeated OVA challenge gradually induced diarrhea and all of the mice in the OVA group developed diarrhea after the 7th OVA challenge (Figure 1). The high dose of PANE (0.1%) markedly attenuated the incidence of diarrhea from the 5th to the last OVA challenge, in which the rate of diarrhea from the 8th and 10th OVA challenge was reduced from 100% to 40% (Figure 1). The induction of allergic diarrhea in mice receiving 0.05% PANE was also significantly attenuated from the 6th to the 8th OVA challenge (Figure 1).

### PANE attenuated intestinal inflammation and mast cells infiltration and degranulation

**Figure 3:**
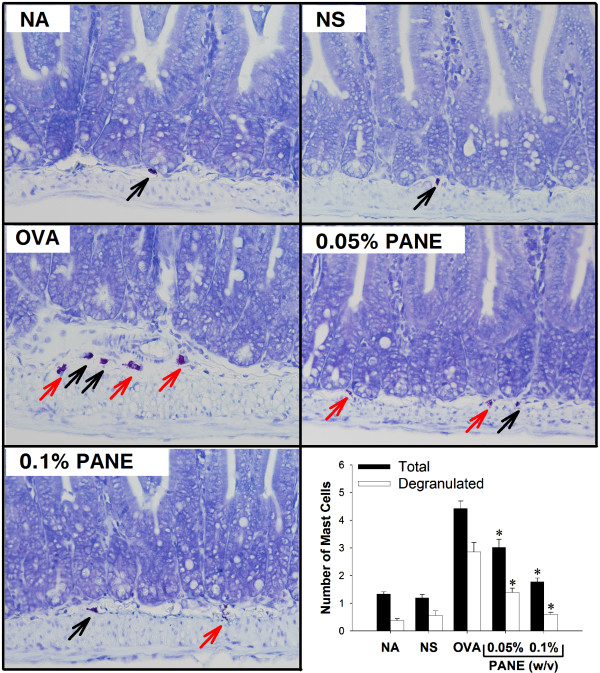
**PANE attenuated the infiltration and degranulation of mast cells in the duodenum.** Tissue sections of the duodenum were stained with toluidine blue. (**A**) Representative sections of the duodenum were shown (original magnification, ×400). Black and red arrows indicate non-degranulated and degranulated mast cells, respectively. (**B**) The number of total and degranulated mast cells was counted manually. Three measurements per section were counted. Data are expressed as mean ± SE of 11 samples per group pooled from 3 independent experiments. ^*^, *p* < 0.05 compared to the OVA group.

We further examined the histological changes associated with food allergy in the duodenum using H&E staining. Compared to the normal morphology in the NS group, the shape of the villus was irregular and short, and the crypt was heavily infiltrated with inflammatory cells in the OVA group, demonstrating strong inflammatory reactions with a reduced villus/crypt ratio associated with food allergy (Figure 2; OVA *vs.* NS). Notably, both doses of PANE prevented the morphological alterations, and restored the reduced villus/crypt ratio in allergic mice (Figure 2; PANE *vs.* OVA). The infiltration and degranulation of mast cells were examined using toluidine blue staining. As shown in Figure 3, both doses of PANE significantly attenuated mast cell infiltration and degranulation in the duodenum of OVA-sensitized and challenged mice.

### PANE attenuated the production of IgE

Mast cells armed with allergen-specific IgE are the effector cells to initiate hypersensitivity responses. We therefore examined if PANE affected the production of total and OVA-specific IgE. A marked increase in the serum level of total and OVA-specific IgE in the OVA group was observed (Table 2; OVA *vs.* NA), which was significantly attenuated in mice treated with both doses of PANE (Table 2).

**Table 2:** PANE attenuated the production of serum total and OVA-specific IgE

**Group**	**Total IgE (ng/mL)**	**OVA-IgE (O.D.)**
NA	211.01 ± 38.38	0.02 ± 0.00
NS	286.83 ± 15.55	0.01 ± 0.00
OVA	4011.59 ± 667.01^#^	0.25 ± 0.06^#^
0.05% PANE	2488.36 ± 356.68^*^	0.11 ± 0.02^*^
0.1% PANE	1956.17 ± 381.27^*^	0.10 ± 0.02^*^

Data are expressed as the mean ± SE of 11 samples pooled from 3 independent experiments. ^#^, *p* < 0.05 compared to the NS group. ^*^, *p* < 0.05 compared to the OVA group.

### PANE attenuated Th2 responses and induced myeloid-derived suppressor cells

**Figure 4:**
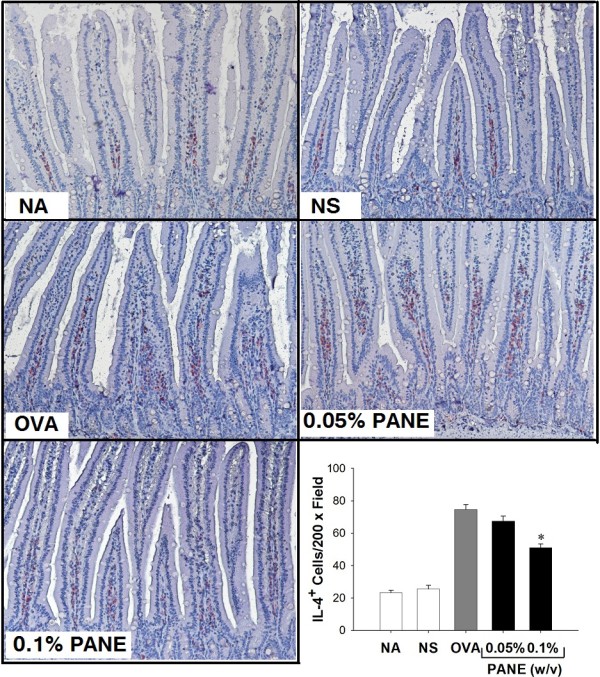
**PANE reduced the number of IL-4**^**+ **^**cells in the duodenum.** Mice were treated as the protocol described in the Methods section. The expression of IL-4 in the duodenum was examined using IHC staining. Representative sections stained with IL-4 were shown (original magnification, x200). Cells with red signals around the blue nuclei indicate the IL-4 positive cells. Six measurements per section were counted. Data are expressed as the mean ± SE of 11 samples per group pooled from 3 independent experiments. ^*^, *p* < 0.05 compared to the VH group.

**Figure 5:**
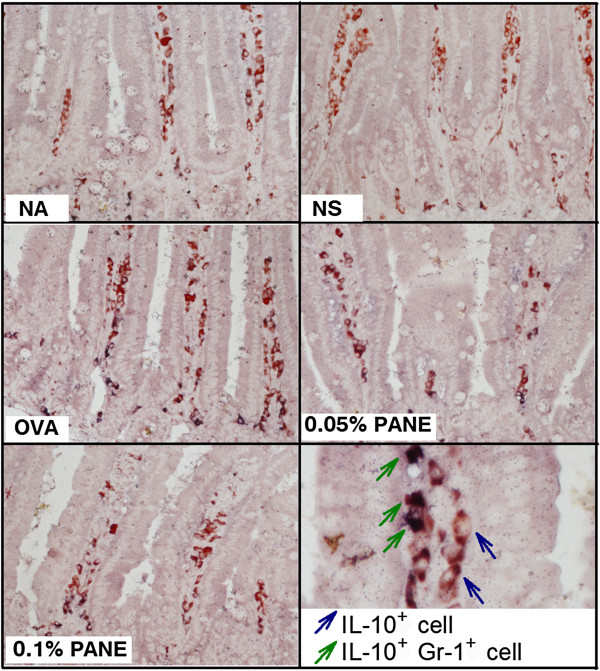
**PANE increased the number of Gr-1**^**+**^**IL-10**^**+ **^**cells in the duodenum.** Mice were treated as the protocol described in the Methods section. Representative sections stained for Gr-1 and IL-10 are shown (original magnification, x 200). Cells with blue arrows indicate IL-10^+^ cells. Green arrows indicate Gr-1^+^IL-10^+^ cells showing red signals around the dark blue nuclei. Quantitative data are shown in Table 3.

We investigated whether PANE affected the expression of IL-4 in the duodenum of allergic mice. Results from IHC staining showed that PANE attenuated the expression of IL-4^+^ cells in the duodenum (Figure 4), whereas the number of IFN-γ^+^ cells was not altered (data not shown). To further address the potential mechanisms for ANE-mediated anti-allergic effects, we examined whether PANE affected the induction of myeloid-dericed suppressor cells (MDSC) in mice with food allergy. The number of Gr-1^+^, IL-10^+^ and Gr-1^+^IL-10^+^ cells was scarce in the NS and OVA groups. Oral intake of PANE significantly increased the number of Gr-1^+^, IL-10^+^ and Gr-1^+^IL-10^+^ cells in the duodenum, as compared to the VH control group (Figure 5; Table 3).

**Table 3:** **PANE induced Gr-1**^**+**^**and Gr-1**^**+**^**IL-10**^**+ **^**cells in the duodenum of allergic mice**

	**Number of positive cells**^***a***^				
	**NA**	**NS**	**OVA**	**PANE**	
				**0.05%**	**0.1%**
Gr-1^+^	2 ± 0	2 ± 0	2 ± 0	2 ± 0	4 ± 0^*^
IL-10^+^	13 ± 1	15 ± 2	24 ± 2^#^	29 ± 2^*^	31 ± 2^*^
Gr-1^+^IL-10^+^	1 ± 0	2 ± 0	2 ± 0	2 ± 0	3 ± 0^*^

^*a*^ The number of single positive cells was quantified using the software Image-Pro Plus 5.1, and double positive cells were counted manually. Data are expressed as mean ± SEM (n = 6). ^#^, *p* < 0.05 compared to the NS group. ^*^, *p* < 0.05 compared to the OVA group. Results are a representative of 3 independent experiments.

## Discussion

Although *Arecae semen* has been used as a traditional Chinese medicine for centuries to treat certain gastrointestinal disorders such as diarrhea [1], it remains unclear if areca-derived constituents affect allergic diarrhea associated with food allergy. As areca nuts contain a rich amount of polyphenols which have been documented to possess immunomodulatory and anti-allergic properties [8,9], the present study aims to investigate the potential effect of areca polyphenols on food allergy. Our results demonstrated that oral intake of PANE attenuated intestinal inflammation, mast cell activation and the occurrence of allergic diarrhea. To the best of our knowledge, these data provide the first evidence to show the anti-food allergic effects of areca-derived polyphenols.

Areca-derived polyphenols contain monomers of (+)-catechin and (−)-epicatechin and their polymerized oligomers that are structurally similar to apple-derived procyanidins [6,16]. Procyanidins are the second most abundant group of natural phenolics widely distributed in the plant kingdom [7-9]. The health-promoting potential of procyanidins has attracted a great deal of attention. Notably, previous studies reported that oral intake of apple procyanidins attenuated atopic dermatitis, food allergy and inflammatory colitis [8,9]. Together with the present results, it is suggested that both apple- and areca-derived procyanidins are functional phytochemicals with potential anti-allergic properties.

The present data showed that oral intake of PANE possess anti-inflammatory activities against food allergy; on the contrary, intraperitoneal injection of PANE induced pro-inflammatory responses [26]. Previous studies showed that procyanidins can be absorbed and detected in the plasma after oral uptake [27,28]. The complex linkage and the mean degree of polymerization (mDP) of procyanidins may affect their absorption, bioavailability and metabolism [29]. For example, the rate of absorption of monomeric to trimeric procyanidins from the gut was 10 times greater than that of highly oligomeric procyanidins (mDP > 6) [30], which are poorly absorbed in the gut and largely reach the colon where they can be degraded into various metabolites by the microflora [31]. Hence, we speculate that the oligomeric procyanidins with high chain-length can enter the systemic circulation system by intraperitoneal injection, while monomer to trimmers may be the major constituents absorbed into the bloodstream after oral administration of PANE. We assumed that the contrasting anti- and pro-inflammatory effects between the present and previous results may be due to the different absorption profile of PANE constituents between oral and intraperitoneal administrations. Further studies are required to address this issue.

We previously reported that intraperitoneal administration of PANE enhanced Th1 cell-mediated immunity and induced the development of myeloid-derived suppressor cells (MDSC) in a murine model of delayed-type hypersensitivity [26]. MDSC have been shown to regulate T-cell homeostasis under various pathophysiological conditions, such as inflammation or cancers [32]. The generation of MDSC is suggested to improve survival associated with graft transplantation by suppressing alloantigen-reactive T-cell reactivity [33]. Moreover, adoptive transfer of MDSC dampened allergen-induced airway inflammation in an IL-10-dependent manner [34]. MDSC may produce IL-10 to promote the generation of regulatory T cells and suppress the expression of FcϵRI and the activation of mast cells to prevent excessive inflammation [35,36]. To date, it remains mostly unknown if MDSC play a role in allergic responses. In the present study, we demonstrated that PANE increased the infiltration of IL-10-producing MDSC in the intestine of mice with food allergy. These results suggest that MDSC may be involved in the regulation of the gut immunobalance, and the anti-allergic effect of PANE may be mediated, at least in part, by the induction of MDSC.

Lamina propria cells consist of approximately 2-3% of mast cells in normal gastrointestinal mucosa, which can augment up to tenfold in disease conditions [37]. Mast cells express high-affinity IgE receptors to bind IgE; re-exposure of allergen triggers the release of inflammatory mediators via crosslinking of surface-bound IgE receptors [38]. Therefore, inhibition of mast cell activation and degranulation is a strategy for managing allergy. Previous studies reported an inhibitory effect of areca nut extracts on mast cell functions *in vitro*[10]. Our data further demonstrated the suppressive effect of PANE on mast cell infiltration and degranulation in mice with food allergy. Furthermore, the serum level of OVA-specific IgE was attenuated by PANE, indicating that PANE down-regulated the function of mast cells and IgE synthesis leading to the attenuation of intestinal inflammation.

## Conclusion

In conclusion, the present study demonstrated the anti-food allergic properties of PANE administered via drinking water in the employed murine model of food allergy. The anti-allergic effect of PANE was associated with the suppression of Th2 immunity and the induction of MDSC in the inflamed site. These data provide evidence to substantiate some of the claimed beneficial effects of *Arecae semen*, in particular anti-diarrhea. The potential of areca-derived polyphenols as functional phytochemicals against food allergy warrants further investigation.

## Competing interests

The authors declared that they have no competing interests.

## Authors’ contributions

CCW carried out experiments and participated in the analysis of data. YRL carried out experiments. MHL supervised animal experiments and participated in the preparation of the manuscript. TRJ designed the study, supervised experiments and refined the manuscript for publication. All authors read and approved the final manuscript.

## Pre-publication history

The pre-publication history for this paper can be accessed here:

http://www.biomedcentral.com/1472-6882/13/154/prepub
